# Interplay between oxidative stress and physical exercise in hospitalized older adults: a secondary analysis of an RCT using malondialdehyde as a biomarker

**DOI:** 10.3389/fragi.2025.1708162

**Published:** 2026-01-05

**Authors:** Chenhui Chenhuichen, Pedro Azanon-Nogueira, Maite Izco-Cubero, Iciar Echeverria-Beistegui, Patricia Alvarez-Rodriguez, Fabíola Zambom-Ferraresi, Fabricio Zambom-Ferraresi, Marisa Fernández González De La Riva, Consuelo Borrás, Nicolas Martínez-Velilla

**Affiliations:** 1 Geriatrics Department, Universitary Hospital of Navarra, Pamplona, Spain; 2 Navarrabiomed, Pamplona, Spain; 3 Instituto de Investigacion Sanitaria de Navarra, Pamplona, Spain; 4 Universidad Publica de Navarra, Pamplona, Spain; 5 MiniAging Research Group, Department of Physiology, Faculty of Medicine, University of Valencia, Institute of Health Research-INCLIVA, Valencia, Spain; 6 Department of Biomedical Sciences, School of Health Sciences, Universidad CEU Cardenal Herrera, Valencia, Spain; 7 Instituto de Salud Carlos III, Centro de Investigacion Biomedica en Red Fragilidad y Envejecimiento Saludable, Madrid, Spain; 8 Geriatrics Department, Universidad de Navarra, Pamplona, Spain

**Keywords:** diabetes, exercise, sex differences, oxidative stress, physical function

## Abstract

**Introduction:**

Oxidative stress, driven by the imbalance between reactive species from oxygen and nitrogen and antioxidant defense mechanisms, plays a pivotal role in aging-related pathologies. Structured multicomponent exercise interventions have mitigated hospital-acquired disability by improving physical and cognitive function and quality of life. However, the underlying molecular mechanisms of this improvement remain partially understood.

**Methods:**

We conducted a secondary analysis of a randomized controlled trial to investigate the impact of a supervised exercise program on oxidative stress in hospitalized older adults. Participants were randomized to a 3-day tailored exercise program based on baseline functional capacity. Serum malondialdehyde (MDA) levels (μmol/mL) and the oxidative oxidation of total proteins (PO) were measured. RESULTS: Seventy-two participants were included in this subanalysis (mean age 86.8 years [SD 4.96], 53.8% female [n = 39]). The exercise group showed a minimal change in MDA levels, while the control group exhibited a significant increase, with a between-group difference of −0.24 μmol/mL (p < 0.01). Subgroup analyses demonstrated significant benefits in patients with diabetes and in women. The intervention improved functional capacity and subjective health status. Participants with lower baseline oxidative stress levels showed greater improvement in SPPB compared to those with higher baseline levels.

**Discussion:**

Structured exercise may mitigate the increase in oxidative stress in hospitalized older adults, particularly in women and those with diabetes. The magnitude of functional improvements could depend on baseline oxidative status, highlighting the need for personalized interventions. Future research should explore long-term effects, biomarkers, and tailored protocols to optimize outcomes in this population.

## 1 Introduction

Hospital admission in older adults represents a critical health event that frequently triggers a cascade of adverse outcomes, with functional decline affecting up to 30%–60% of older patients (Loyd et al., 2020). This deterioration is driven by complex pathophysiological mechanisms, including increased oxidative stress, inflammation, worsening oral intake, and prolonged immobility (Bagnato et al., 2025; Wakabayashi and Sashika, 2014). These factors influence the disease prognosis and lead to reduced muscle mass, impaired mobility, deteriorating cognitive function, and diminished quality of life (Sprung et al., 2021; López Jiménez et al., 2024). The intersection between physical inactivity during hospitalization and age-related oxidative damage presents a compelling target for therapeutic interventions aimed at mitigating these adverse effects (Barrera et al., 2018).

Oxidative stress, characterized by an imbalance between the production of reactive species derived from oxygen (ROS) and nitrogen (RNS) and the body’s antioxidant defense mechanisms, plays a pivotal role in aging-related pathologies and functional decline (Forman and Zhang, 2021). Under physiological conditions, low levels of oxidative stress support the essential redox signaling pathways that regulate cellular functions. However, excessive ROS/RNS production produces a toxic range, causing oxidative damage to lipids, proteins, and DNA (Luo et al., 2020). Malondialdehyde (MDA), a by-product of polyunsaturated fatty acid peroxidation and a representative marker of lipid peroxidation in combination with protein oxidation, a diverse process characterized by the modification of amino acid residues, serves as a reliable biomarker for quantifying oxidative stress (Mas-Bargues et al., 2021). Elevated MDA levels have been documented in various clinical contexts, including in patients with different types of cancer (Jelic et al., 2021) and, in critical care settings, higher MDA levels have been associated with the severity and mortality of patients with sepsis (Lorente et al., 2013). Protein oxidation (PO) has been correlated to sarcopenia and all-cause mortality in patients undergoing hemodialysis (Song et al., 2020) and poor grip strength in older women (Howard et al., 2007). Globally, increased oxidative stress in older adults is related to cardiovascular and neurodegenerative diseases (Liguori et al., 2018), increased mortality risk (Semba et al., 2007) and worse clinical outcomes during hospitalization (Vazquez-Agra et al., 2022).

While structured exercise interventions have demonstrated efficacy in preventing functional decline in hospitalized older adults (Martínez-Velilla et al., 2019), the underlying molecular mechanisms remain incompletely understood. Furthermore, there is a significant knowledge gap regarding how these responses differ by sex and among individuals with varying comorbidities. Evidence from previous studies indicates that the effects of physical exercise on oxidative stress depend on the type, intensity, and duration of the activity. Whereas acute bouts of prolonged, high-intensity exercise tend to elevate oxidative stress levels, short-to long-term sessions (5–12 days) appear to enhance antioxidant capacity (Powers et al., 2020).

This secondary analysis of a randomized controlled trial aimed to evaluate the relationship between a structured exercise program and oxidative stress in hospitalized older adult patients, focusing on the influence of sex and diabetes. We hypothesized that a structured exercise intervention during hospitalization would favorably modulate oxidative stress markers, with potential variations in response patterns based on patients’ baseline characteristics.

## 2 Methods

This is a secondary analysis of a randomized controlled trial (RCT). We conducted a parallel-group randomized controlled trial between December 2020 and September 2022 in the acute care unit of a tertiary hospital. The institutional ethics committee approved the protocol (reference number: Pyto 2018/7), and all the participants provided written informed consent. The inclusion criteria were as follows: age ≥75 years, independent ambulation pre-hospitalization (with or without assistive devices), sufficient cognitive status to obey verbal commands (MMSE >18), and an expected hospital stay >72 h. The exclusion criteria comprised terminal illness, acute cerebrovascular accident, hemodynamic instability, or contraindications to performing physical exercise.

### 2.1 Randomization and blinding

Participants were randomized 1:1 to the intervention or control groups using computer-generated random numbers with concealed allocation. Because of the nature of the intervention, complete blinding was unfeasible, but outcome assessors remained blinded to group allocation.

### 2.2 Intervention protocol

The intervention involved a multicomponent, progressive exercise program delivered daily in the mornings over three consecutive days (including the weekends). Adapted from the Vivifrail multicomponent program designed specifically for older adults (Izquierdo et al., 2019), the morning session included progressive resistance, balance, and walking exercises tailored to the individual’s functional capacity using variable resistance training machines, with two to three sets of eight to ten repetitions performed with a load equivalent to 50%–70% of their one-repetition maximum at a rapid intentional velocity. The resistance exercise targeted major muscle groups, including the lower limbs (chair squats, leg press, and bilateral knee extension) and the upper body (seated chest press). The intervention group was encouraged to take daily walks in the hallway during the afternoon. Adherence was documented using standardized forms, and adverse events were systematically recorded. The control group received standard hospital care, including routine mobilization as prescribed by the attending physician, but no structured exercise program.

### 2.3 Blood sampling

After an 8-h to 10-h fast, a blood sample was obtained between 6 and 7 a.m. from an antecubital vein. Blood samples were drawn into EDTA tubes on the same day of the first assessment (baseline; 1–2 days after admission) and 24 h after the last bout of exercise (discharge assessment). The samples were centrifuged for 15 min at 3,000 rpm at room temperature. The plasma was obtained, transferred to an Eppendorf tube, and stored at −80 °C, after which MDA concentrations and protein oxidation were analyzed.

### 2.4 Outcomes

The primary outcomes were serum MDA levels and protein oxidation (PO). MDA levels (μmol/mL), measured at baseline and discharge using high-performance liquid chromatography, following a modified protocol (Mas-Bargues et al., 2021) based on that described by Wong et al. (1987). This particular and sensitive protocol distinguishes MDA concentration from other aldehydes that may react with 2-thiobarbituric acid. Briefly, this method is based on the reaction of one MDA molecule with two molecules of thiobarbituric acid (TBA), producing a pink chromogen with absorbance at 532–536 nm. A standard curve was established to identify and quantify the MDA in the analyzed samples. Oxidative oxidation of total proteins (PO) was assessed by immunoblot detection of plasma protein carbonyl groups using the OxyBlot Protein Oxidation Detection kit (Millipore, Billerica, MA) according to the manufacturer’s instructions. Briefly, it was determined by analyzing carbonyl groups in protein side chains, which were derivatized to 2,4-dinitrophenylhydrazone via reaction with 2,4-dinitrophenylhydrazine, enabling detection by Western blotting with specific antibodies. The procedure to quantify total protein carbonyls using the OxyBlot kit involved densitometry of the OxyBlot and Ponceau staining, followed by calculating the ratio of the total density in the OxyBlot to that in the Ponceau.

Secondary outcomes included physical performance, assessed using the Short Physical Performance Battery (SPPB), a validated test that evaluates physical function through balance, gait speed, and chair stand tests (Guralnik et al., 1994). Muscular strength was measured in the primary trained muscle groups: leg press, chest press, and knee extension, and quality of life was measured using the Visual Analogue Scale (EuroQol-VAS).

### 2.5 Statistical analysis

Data are presented as mean ± 95% confidence interval (95% CI) and standard deviation (SD), or as number (proportion) for primary outcomes. Normality was assessed using the Shapiro-Wilk test. Baseline data were compared between groups using independent t-tests (for continuous variables) or Chi-squared (for categorical variables). An analysis of covariance (ANCOVA) was performed to compare post-intervention values between groups, adjusting for baseline measures, age, and sex. Spearman’s rank correlation coefficients were calculated to assess possible associations between variables. Additional analyses included dividing baseline oxidative stress values into quartiles and creating interaction terms between intervention and these quartiles for regression models. A combined oxidative stress variable was generated by log-transforming and standardizing baseline MDA and PO values, which were then summed to obtain a single composite score. Subgroup analyses were also performed based on whether baseline values were above or below the mean.

## 3 Results

Among the 90 patients included in the original randomized controlled trial, blood samples were obtained from only 72 participants (mean age 86.85 ± 4.96 years, 54.2% female), with well-balanced baseline characteristics across groups. The overall prevalence of diabetes was 29.23% (intervention: 35.3% vs. control: 28.9%). The baseline characteristics are presented in Table 1.

**TABLE 1:** Baseline characteristics^a^.

​	Control (n = 36)	Intervention (n = 36)
Age (years)	87.0 (4.77)	86.69 (5.21)
Female^b^	20 (55.6%)	19 (52.8%)
Barthel index	91.11 (8.87)	91.29 (10.39)
Mini-mental state examination	24.39 (4.32)	24.15 (5.05)
Cumulative illness rating scale-geriatric	11.43 (4.15)	10.94 (4.16)
Short physical performance status (SPPB)	6.25 (2.89)	5.69 (2.71)
Handgrip (kg)	18.84 (7.70)	19.41 (6.26)
EuroQol visual analogue scale	70.94 (19.59)	64.17 (20.75)

^a^ No statistically significant differences between-groups were found. Data are presented as mean (SD), unless stated differently.

^b^ Data are expressed as n (%).

**FIGURE 1:**
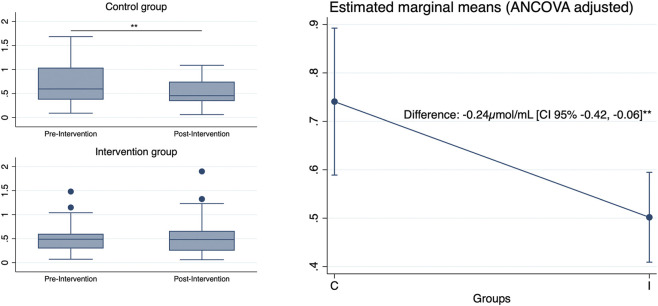
Global changes in MDA levels. On the left, unadjusted raw MDA levels of both groups, pre- and post-intervention, are shown as boxplots representing the interquartile range (25th–75th percentiles) and median, with whiskers extending to the minimum and maximum values within 1.5 × IQR. On the right, estimated marginal means are shown (ANCOVA-adjusted controlling for baseline MDA levels, age, and gender), with error bars representing 95% confidence intervals. C: control group. I: intervention group. Level of significance: *p < 0.05; **p < 0.01.

**FIGURE 2:**
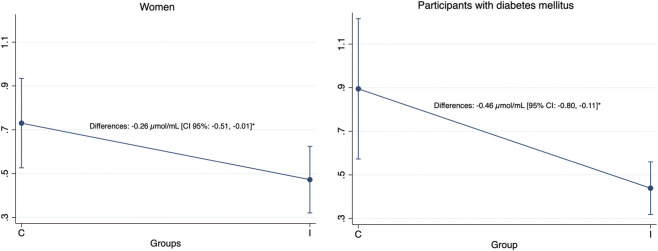
Adjusted group differences in MDA levels according to sex and diabetes mellitus. Estimated marginal means of participants with diabetes (ANCOVA-adjusted controlling for baseline MDA levels, age, and gender), with error bars representing 95% confidence intervals. Values on the left correspond to women, and values on the right correspond to men. C: control group. I: intervention group. Level of significance: *p < 0.05; **p < 0.01.

The exercise intervention significantly affected MDA levels compared to the control (between-group difference: −0.24 μmol/mL [95% CI: −0.42 to −0.06], p < 0.01), with the control group showing a mean increase of 0.22 μmol/mL (95% CI: 0.06 to 0.37, p < 0.01) versus a non-significant minimal change in the intervention group (Δ −0.05 μmol/mL [95% CI: −0.17 to 0.07], p = 0.41) (Figure 1). Subgroup analyses revealed distinct response patterns in patients with diabetes and women (Figure 2). In participants with diabetes, the change in MDA levels between groups was significantly different (−0.46 μmol/mL [95% CI: −0.80 to −0.11], p < 0.05), but this change was not significant in participants without diabetes (−0.14 μmol/mL [95% CI: −0.35 to 0.07], p = 0.18). Similarly, the control women showed a significant increase in MDA levels (Δ 0.21 μmol/mL [95% CI: 0.01 to 0.41], p < 0.05), and there was a significantly different change from baseline to post-intervention between groups (−0.26 μmol/mL [95% CI: −0.51 to −0.01], p < 0.05), while no effect was observed in MDA levels in men. We did not find any significant changes in PO levels after the intervention.

Regarding the secondary outcomes, the intervention improved post-intervention SPPB (between-group difference 1.24 [95% CI: 0.45–2.04], p < 0.01) and the assessed strength parameters: leg press (8.34 [95% CI: 3.09–13.58], p < 0.01), knee extension (4.75 [95% CI: 1.58–7.92], p < 0.01), and chest press (3.19 [95% CI: 1.33–5.05], p < 0.01). Subjective health status (EQ-VAS) also improved after the intervention (9.0 [95% CI: 0.31–17.69], p < 0.04). Subgroup analysis showed that this response differed by gender. While changes in knee extension strength and subjective health status were significantly different only in women, the chest press was significantly different only in men.

Correlation analyses did not reveal any significant relationship between changes in MDA or PO and strength and functional parameters after applying the Benjamini–Hochberg FDR correction, as shown in Table 2. When examining the effect of baseline oxidative stress levels on post-intervention SPPB, after adjusting for age, sex, and baseline SPPB, we found a significant interaction between the intervention and the best baseline oxidative stress levels quartile (ß = 1.95, p = 0.03, *R* ^2^ = 0.71). Moreover, in the subgroup analyses, the intervention effect was not significant among participants with higher oxidative stress (n = 12, ß = 2.81, p = 0.20, *R* ^2^ = 0.75). Still, it remained significant in those with lower oxidative stress (n = 59, ß = 1.14, p = 0.01, *R* ^2^ = 0.69).

**TABLE 2:** Correlation analysis.

Oxidative stress variable (change)	Functional variable (change)	ρ (rho)	p-value	p-value BH
MDA	SPPB	0.13	0.30	0.71
	Handgrip	0.08	0.50	0.71
	Chest press	−0.14	0.27	0.71
	Leg press	−0.09	0.50	0.71
	Knee extension	−0.26	0.04	0.38
PO	SPPB	−0.02	0.88	0.95
	Handgrip	0.03	0.80	0.95
	Chest press	−0.10	0.44	0.71
	Leg press	0.08	0.95	0.95
	Knee extension	0.15	0.24	0.71

Results of Spearman’s correlation analysis, adjusted for multiple comparisons using the Benjamini–Hochberg FDR, method. MDA, malondialdehyde; PO, protein oxidation.

The intervention achieved an 89% adherence rate, with no serious adverse events reported during the exercise sessions. The financial resources allocated to the intervention comprised the part-time employment of a specialized physiotherapist for the study’s duration and an initial capital investment of €5,500 for the acquisition of resistance training equipment.

## 4 Discussion

This secondary analysis of a randomized controlled trial provides evidence that structured exercise during hospitalization may modulate oxidative stress in older adult patients, as indicated by changes in MDA levels. The exercise intervention attenuated the significant increase in oxidative stress observed in the control group, suggesting that supervised multicomponent, physical exercise during hospitalization can counteract the detrimental effects of acute illness and hospitalization on redox balance. This modulation represents a biologically significant change, comparable to other antioxidant interventions reported in previous studies (Mahjabeen et al., 2022; Zamani et al., 2020). In the present 3-day multicomponent adapted exercise intervention, no significant increases in MDA levels were detected 24 h after the final exercise session in the intervention group. This finding supports the notion that adaptive responses in oxidative stress biomarkers may vary not only according to the type and duration of exercise performed but also with the timing of sample collection (Thirupathi et al., 2021; Ceci et al., 2014).

The differential response patterns observed in women and patients with diabetes suggest complex interactions between metabolic status and sex on treatment outcomes and redox balance. We observed a significant between-group difference, suggesting that exercise may confer greater benefits in modulating oxidative stress among patients with diabetes, likely due to their higher oxidative burden. Oxidative stress has been proposed as a key contributor to the pathophysiology of diabetes and its associated complications. At the same time, chronic hyperglycemia promotes the overproduction of reactive oxygen species and depletion of antioxidant defenses (Ighodaro, 2018). In the present study, this imbalance, exacerbated by hospitalization and acute conditions, can be modulated by physical exercise. These results underscore the need for further research on the mechanisms underlying oxidative stress regulation in patients with diabetes.

Furthermore, sex-based differences highlight the need for additional investigations. No significant changes in oxidative stress were observed in men. In contrast, women in the control group exhibited a significant increase in oxidative stress, which was attenuated by exercise in the intervention group. This suggests that women may benefit more from physical activity during hospitalization. Previous studies have reported lower levels of oxidative stress in young women than in men, a relationship that reverses after menopause. At this point, the decline in estrogen levels is one of the primary explanations for the increased oxidative stress found in post-menopausal women (Borrás et al., 2021). These sex differences have been proposed as a factor in the differential risk of certain diseases between sexes (Tenkorang et al., 2018). However, further research is needed to elucidate the underlying mechanisms of this topic and to determine whether specific training programs can more effectively modulate oxidative stress. These findings emphasize the importance of considering sex as a critical variable when designing interventions for older adults.

Additionally, the present findings suggest that baseline oxidative stress levels may influence the functional response to exercise during hospitalization. Participants with lower baseline oxidative stress levels showed greater improvements in physical performance, as measured by the SPPB, while those with elevated oxidative stress showed a limited response. This pattern may indicate that redox homeostasis modulates physiological adaptations to exercise, consistent with previous research showing that excessive oxidative stress may attenuate functional gains following physical interventions (Margaritelis et al., 2020).

Collectively, these findings emphasize the modulatory effect of oxidative stress on a physical exercise intervention and its therapeutic potential to mitigate it and preserve functional capacity in hospitalized older adults. However, the heterogeneity in response patterns underscores the need for personalized approaches that account for individual factors, such as comorbidities and sex-based differences. Future studies should explore the molecular pathways driving these differential responses, including the role of antioxidant systems and inflammatory markers, to optimize exercise recommendations. By tailoring interventions to individual patient profiles, clinicians can enhance the efficacy of exercise programs, reduce the risk of functional decline, and improve clinical outcomes in this vulnerable population, ultimately contributing to better recovery and quality of life after hospitalization.

Our findings extend the current knowledge in several important ways. They not only demonstrate that oxidative stress modulation through exercise is feasible even in the challenging context of acute hospitalization but also highlight the importance of identifying specific subgroups that may require tailored interventions. Moreover, it confirms the potential of short-term programs to enhance functional capacity in hospitalized older adults. However, as previously suggested, a 4-day exercise program may be optimal for enhancing functional and cognitive capacities (Sáez de Asteasu et al., 2024).

The observed MDA modulation likely reflects multiple underlying mechanisms of action. Exercise-induced activation of antioxidant defense systems, including upregulation of superoxide dismutase and glutathione peroxidase, may play central roles (Powers et al., 2022). Additionally, improving mitochondrial function associated with physical activity could reduce ROS production (Hood et al., 2019).

### 4.1 Strengths, limitations, and future directions

This study had several strengths, including its randomized controlled design with adequate statistical power, comprehensive functional outcome assessments, detailed subgroup analyses, a notable intervention adherence rate (89%), and standardized protocols for measuring oxidative stress biomarkers. However, significant limitations must be acknowledged: the single-center nature of the study potentially limits external validity, the inherent inability to blind participants to the exercise intervention, considerable variability in hospitalization duration among participants, a limited number of diabetic participants and participants with high levels of baseline oxidative stress, and the absence of correlations between changes in these markers and the physical benefits observed. These limitations inform our proposed future research directions, which should prioritize multicenter, long-term follow-up studies to evaluate the durability of oxidative stress modulation, expand the investigation to include additional oxidative stress biomarkers and inflammatory mediators, develop and assess modified exercise protocols tailored explicitly for different comorbidities. Future exercise protocols should be adjusted for baseline oxidative stress and sex, and mechanistic studies should be conducted to elucidate the pathways through which exercise modulates oxidative stress in specific subgroups. Moreover, future studies should explore the potential synergistic effects of combined interventions incorporating both exercise and antioxidant supplementation, as evidence in younger populations suggests that such supplementation may modulate exercise-induced muscle damage and enhance recovery (Canals-Garzón et al., 2022). Finally, future trials should evaluate the cost-effectiveness of implementing hospital-based exercise programs. Integrating these future research priorities will be crucial for advancing our understanding of how to modulate exercise-induced oxidative stress in hospitalized older adults and for optimizing intervention protocols across different patient subgroups.

## 5 Conclusion

This randomized controlled trial suggests that a structured exercise program during hospitalization may help modulate oxidative stress in older women and older people with diabetes, as measured by serum MDA levels. Moreover, baseline oxidative stress levels could help predict the functional response to exercise. These findings support the need for future studies to evaluate this effect further and to design more individualized approaches. The intervention’s effectiveness across different profiles of complex older patients and its safety profile support its broad applicability in acute-care settings.

## Data Availability

The raw data supporting the conclusions of this article will be made available by the authors, without undue reservation.
